# The Use of Telemedicine in Cancer Clinical Trials: Connect-Patient-to-Doctor Prospective Study

**DOI:** 10.2196/31255

**Published:** 2022-01-27

**Authors:** Yasmine Meghiref, Charles Parnot, Claire Duverger, Françoise Lilly Difoum, Audrey Gourden, Halima Yssaad, Caroline Leiterer, Caroline Bedekovic, Julien Blanchard, Houria Nait Ammar, Antoine Schernberg, Hélène Vanquaethem, Carole Helissey

**Affiliations:** 1 Bégin Military Teaching Hospital Saint-Mandé France; 2 Cureety Dinan France

**Keywords:** telemedicine, clinical trial, neoplasms, patient-reported outcome measures

## Abstract

**Background:**

Telemedicine is currently being adopted for the management of patients in routine care. However, its use remains limited in the context of clinical trials.

**Objective:**

This study aimed to demonstrate the feasibility of telemonitoring and patient-reported outcomes collection in the context of clinical trials.

**Methods:**

The patients who were included in an interventional oncology clinical trial were eligible. The patients were registered with a digital tool to respond to a patient-reported outcomes questionnaire (ePRO) based on CTCAE (The Common Terminology Criteria for Adverse Events, National Cancer Institute), version 5.0, personalized to their pathology and treatment. An algorithm evaluated the health status of the patient based on the reported adverse events, with a classification in 4 different states (correct, compromise, state to be monitored, or critical state). The main objective was to evaluate the feasibility of remote monitoring via a connected platform of patients included in a clinical trial.

**Results:**

From July 1, 2020, to March 31, 2021, 39 patients were included. The median age was 71 years (range 41-94); 74% (n=29) were male, and 59% (n=23) had metastatic disease. Out of the 969 ePRO questionnaires completed over the course of the study, 77.0% (n=746) were classified as “correct,” 10.9% (n=106) as “compromised,” and 12.1% (n=117) as “to be monitored” or “critical.” The median response time was 7 days (IQR 7-15.5), and 76% (25/33) of the patients were compliant. Out of the 35 patients who answered a satisfaction questionnaire, 95% (n=33) were satisfied or very satisfied with the tool, and 85% (n=30) were satisfied with their relationship with the health care team. There were 5 unscheduled hospitalizations during the study period.

**Conclusions:**

Remote monitoring in clinical trials is feasible, with a high level of patient participation and satisfaction. It benefits patients, but it also ensures the high quality of the trial through the early management of adverse events and better knowledge of the tolerance profile of experimental treatments. This e-technology will likely be deployed more widely in our clinical trials.

## Introduction

### Remote Monitoring of Cancer Patients

Telemedicine has been shown to provide a level of care quality at least equivalent to in-person care, with high levels of patient and health care professional satisfaction [1]. The advantages of remote monitoring of patients are the following: early and real time detection of illnesses, ability to continuously monitor patients, prevention of worsening of illnesses and untimely deaths, cost reduction in hospitalizations, reduction in the number of hospitalizations, more accurate readings without interfering with the daily activities of patients, improved efficiency of health care services through the use of digital communication, emergency medical care, service for patients with mobility issues, emergency care for traffic accidents and other injuries, and usage of noninvasive medical interventions [2]. The collection of patient-reported outcomes (PRO) using a telemonitoring approach results in an evaluation closer to the patient’s experience of the disease, allowing adjustments to the treatment in order to improve tolerance and compliance. This also improves communication between the health practitioner and the patient [3].

In France alone, 382,000 new cases of cancer are diagnosed every year, and the number of cancer deaths is estimated at 157,400 [4]. Cancer remains a serious public health problem. The use of remote monitoring in the care of cancer patients has shown a significant reduction in mortality compared with standard care [5]. Despite the known benefits, its implementation and use in clinical trials remain limited. Barely 50% of clinical trials in oncology assess the perception of patients, and only 20% of published trials report quality-of-life data and PRO. This figure drops drastically if the study is negative [6,7]. There are various explanations for the lack of such data, including the difficulty in using the existing tools and interpreting their output, as well as the lack of training of the medical teams.

Clinical trials are a critical tool to evaluate new approaches for screening, diagnosis, treatment, and patient care improvements. For drug development, the results of these trials are mandatory for regulatory approval and provide clinicians with new strategies based on efficacy and safety data. Thus, the lack of PRO in the context of clinical trials means that highly relevant information for decision-making is often unavailable to patients, oncologists, and policy makers.

This connect-patient-to-doctor study aimed to demonstrate the feasibility of telemonitoring and PRO collection in the context of clinical trials. It was conducted at the Bégin Military Teaching Hospital, which typically participates in 30 clinical trials every year that include around 50-60 new patients. The primary hypothesis of this paper was that we should see a high level of compliance with the use of the telemonitoring platform, which would thus be a useful complementary tool in the care of the patients, resulting in a better understanding of drug safety.

## Methods

This study is a prospective study, conducted in Clinical Research Unit of Bégin Military Teaching Hospital. It was declared to the National Institute for Health (Institut National des Données de Santé, Data MR) and was reported to France’s National Commission on Informatics and Liberty, reference 2222625.

### Patients

The study ran from July 1, 2020, until March 31, 2021, and included 39 patients. Any patient who was treated at the Bégin Military Teaching Hospital and was included in an interventional oncology clinical trial was eligible for the study. All trials were considered for inclusion, regardless of their phase and promoter type (academic or industrial). There were 2 exclusion criteria for our study: patients who did not agree to use a digital telemonitoring tool and minors (17 years old or less). Patients were included at the time of a hospital visit as long as they were receiving an antitumoral treatment, regardless of the starting date of the clinical trial. Patients with internet access via their smartphone or via a computer were included in the “telemonitoring” cohort. Patients without internet access or with little autonomy from the tool were included in the “call session” cohort and were contacted by telephone at regular intervals. All the patients included in the study signed a consent for this trial.

### Study Design

Each cancer patient was allowed to respond to a symptomatology questionnaire personalized to their pathology and treatment. The various symptomatology questionnaires used in the study were created by a pluridisciplinary team of oncologists working with the Cureety team. Each questionnaire includes questions for 10 to 20 adverse events relevant to a specific pathology and treatment. The individual questions follow the CTCAE (Common Terminology Criteria for Adverse Events) grading for each adverse event and mostly use the phrasing of the PRO-CTCAE questions and list of prewritten answers (single-select multiple-choice question); however, they also include some modifications to allow a more objective grading directly by the patient without requiring further evaluation by a health practitioner, making the CTCAE standard usable as part of this digital monitoring tool.

The patients were introduced to and enrolled into the telemonitoring platform by their medical team who also assigned an appropriate questionnaire depending on the patient pathology and treatment. The patients in the telemonitoring cohort were then fully autonomous in the use of the platform, with an initial email that allowed them to create their credentials, followed by an information panel in the web application on their first login; the patients were then free to answer the symptomatology questionnaire as often as they wanted (up to once a day), and would otherwise receive text message reminders every 1 or 2 weeks depending on the questionnaire (see below) with a link to the web application. Patients in the call session cohort were called by the medical team once a week, who went over the questionnaire over the phone if the patient was available and willing to answer. All patients were also free to contact the medical team at any time over the phone or via email. More generally, they were clearly instructed that the telemonitoring tool was not meant to replace more traditional care practices, only to supplement them.

**Figure 1:**
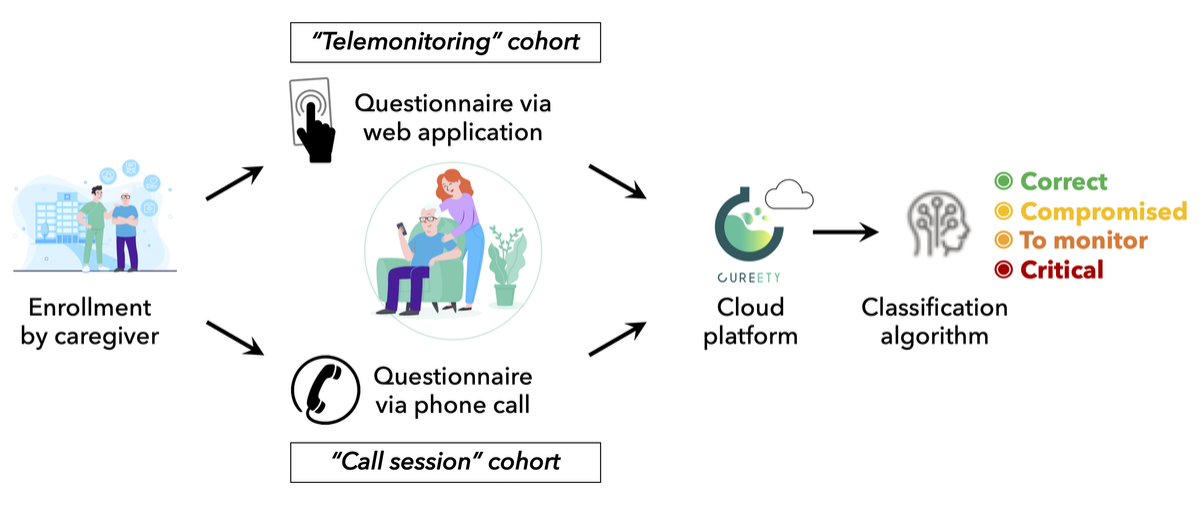
Cancer patient care using the connected telemonitoring platform, Cureety.

Each reported adverse event (AE) was classified based on CTCAE, version 5.0. For each completed questionnaire, a global health score was computed by an algorithm that weighed the grades of the reported AEs according to their potential severity for the given pathology and treatment. The score was then used to classify the patient into 1 of 4 different states: correct (green), compromised (yellow), to be monitored (orange), or critical (red) (Figure 1).

At the end of the questionnaire, and for each declared AE, the patient received therapeutic recommendations accordingly. In the case of green or yellow states, the patient received only these therapeutic recommendations. In the case of orange or red states, the patients received the therapeutic recommendations and were also invited to call the hospital or their general practitioner. The clinical research unit’s team also received by email an alert for orange and red states. Patients could contact the hospital team at any time if they needed to.

The primary end point was to assess the feasibility of monitoring cancer patients included in clinical trials, using the connected platform. The patients were expected to respond to their questionnaire at least once a week for any treatment that included chemotherapy or immunotherapy, and otherwise every 2 weeks (hormonotherapy, targeted therapy, and radiotherapy, alone or in combination with each other). The patients were thus considered to be compliant if the median frequency of their responses was below this target, with an extra tolerance of 2 days to take into account acceptable compliance gaps. Compliance was only assessed for patients who were monitored for at least 30 days to ensure enough data had been collected to calculate the frequency of their reports.

The secondary end point was to assess the patient’s tolerance profile during the study, the patient’s satisfaction, and the number of unscheduled hospitalizations. To evaluate satisfaction, at the end of the study, all patients had to complete a satisfaction questionnaire, which contained 8 questions with a 5-level Likert scale for the responses: “strongly disagree,” “disagree,” “neither agree nor disagree,” “agree,” and “strongly agree.” A final open-ended question also allowed the patient to leave additional comments and provide suggestions about the platform.

### Data Collection and Measurements

We collected demographic data (age at inclusion, sex, and comorbidities), disease characteristics (primitive, histology, and stage at inclusion), phase of clinical trial, and type of treatment received. The individual AEs, the grades reported by the patients, as well as the global health status were collected throughout the duration of the clinical trials in which the patients participated. The number of unscheduled hospitalizations was collected from the patient medical records.

### Statistical Analysis

The PRO data (AEs and health status) were collected digitally and entered directly into the Cureety platform database for both the “telemonitoring” cohort (via the patient application), or the “call session” cohort (via the caregiver application, while on the phone with the patient), eschewing the need for a paper questionnaire and ensuring higher data integrity. The entirety of the digital tool including the web application, the cloud server collecting the data, and the classification algorithm running on that server are developed and managed by the Cureety company. Because it hosts health data of patients in France, the entirety of the technical stack is compliant with the “Hébergeur de données de santé” (health data storage) regulation, which encompasses the ISO (international information security standard) 27001 norm, together with additional rules, and ensures stringent security constraints are in place to protect the patient data. To access the platform, the patients had to create an account and use a username and password combination.

The data were then extracted, analyzed, and formatted using Python (Python Software Foundation) scripts. For descriptive data, median and interquartile range (minimum and maximum) were also indicated.

## Results

### Characteristics of the Patients Included in the Study

**Figure 2:**
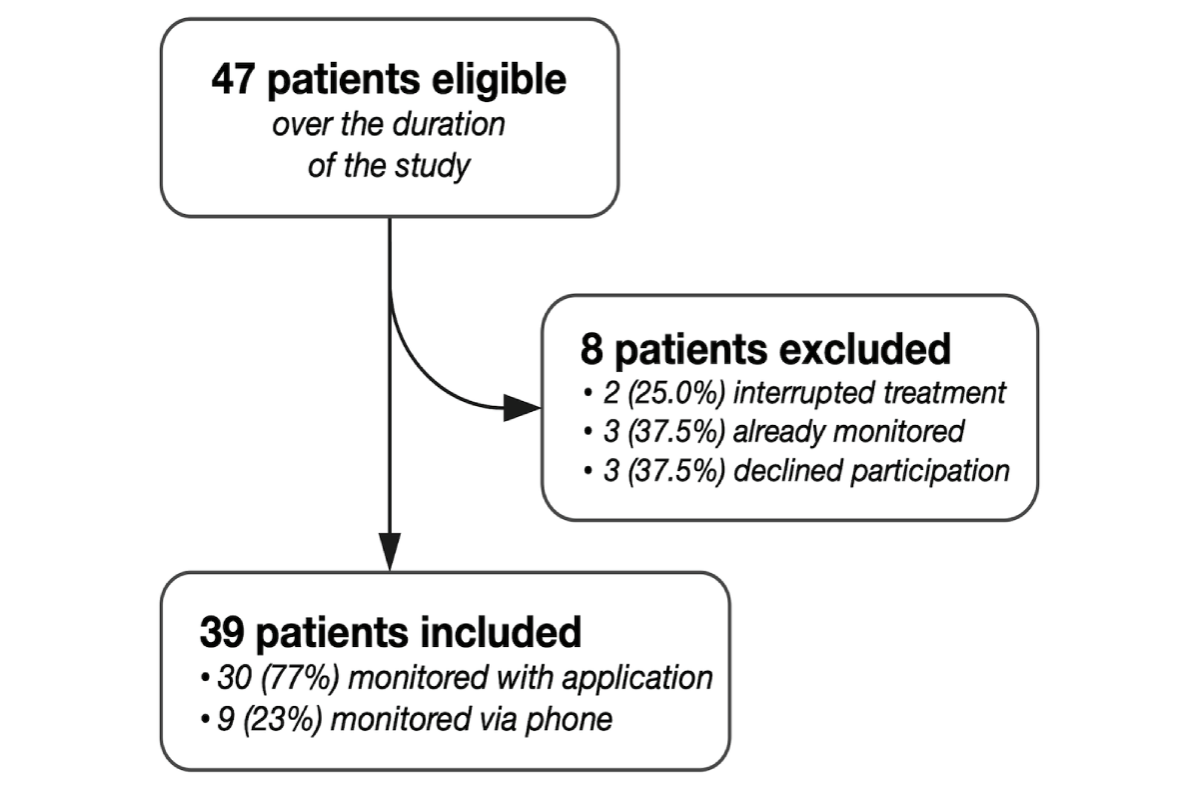
Study flow diagram.

A total of 39 patients were included in our study between July 1, 2020, and March 31, 2021, including 9 in the call session cohort (Figure 2). The median age was 71 years (range 41-94), 74% (n=29) were male, and 69.2% (n=27) presented at least one comorbidity. There was a broad range of primary tumors including prostate cancer (n=23), lung cancer (n=12), breast cancer (n=3), and bladder cancer (n=1). Moreover, 59% (n=23) of the patients had a metastatic disease.

The patients were included in clinical trials in phase III (30, 76.9%), phase II/III (3, 7.7%), phase I (3/39, 7.7%), phase II (2/39, 5.1%) and phase I/II (1/39, 2.6%). There was a broad range of treatment, primarily chemotherapy alone (3/39, 7.7%), chemotherapy with immunotherapy (5/39, 12.8%), new generation of hormonotherapy (11/39, 28.2%), immunotherapy with targeted therapy including PARP (poly adenosine diphosphate-ribose polymerase) inhibitors (3/39, 7.7%), and immunotherapy alone (1/39, 2.6%). Baseline characteristics are summarized in Table 1.

**Table 1:** Baseline characteristics of patients.

Variables			Values
Median age (years), range			71, 41-94
Patients, N (%)			39 (100)
**Gender, n (%)**			
	Female	10 (25.6)	
	Male	29 (74.4)	
**Comorbidities, n (%)**			
	Cardiovascular	13 (33.3)	
	Renal failure	1 (2.6)	
	Chronic obstructive pulmonary disease	1 (2.5)	
	Others	12 (30.8)	
	None	12 (30.8)	
**Location of cancer,** **n (%)**			
	Prostate	23 (59)	
	Lung	12 (30.7)	
	Breast	3 (7.7)	
	Bladder	1 (2.6)	
**Stage, n** **(%)**			
	Metastatic	23 (59)	
	Localized	12 (30.8)	
	Localized advanced	2 (5.1)	
	Oligometastatic	2 (5.1)	
**Clinical trial phase, n (%)**			
	I	3 (7.7)	
	I/II	1 (2.6)	
	II	2 (5.1)	
	II/III	3 (7.7)	
	III	30 (76.9)	
**Type of treatment, n (%)**			
	Chemotherapy and immunotherapy	5 (12.8)	
	Chemotherapy	3 (7.7)	
	New generation of hormonotherapy	11 (28.2)	
	Hormonotherapy and radiotherapy	4 (10.2)	
	Hormonotherapy and targeted therapy	3 (7.7)	
	Immunotherapy	1 (2.6)	
	Immunotherapy and targeted therapy	3 (7.7)	
	Targeted therapy	3 (7.7)	
	Conjugated antibody	1 (2.6)	
	Chemotherapy and hormonotherapy	2 (5.1)	
	Other: adapted physical activity	3 (7.7)	

### Patient-Reported Outcomes on Adverse Events

**Figure 3:**
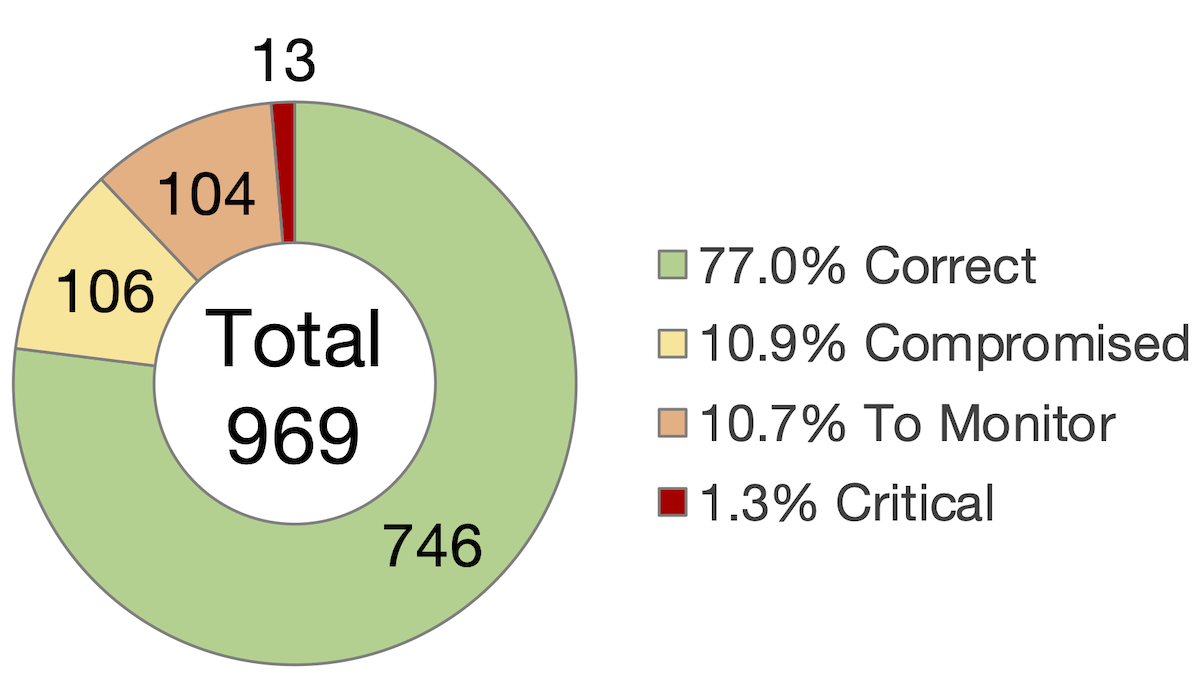
Distribution of health status for the 969 questionnaires completed by the patients over the duration of the study.

Out of the 969 ePRO (patient-reported outcomes questionnaires) completed by the patients, 77.0% (n=746) corresponded to a “correct” state, 10.9% (n=106) to a “compromised” state, 10.7% (n=104) to a state “to be monitored,” and 1.3% to a “critical” state (n=13), as shown in Figure 3. These questionnaires correspond to 15,042 AE questions answered, among which there were 84 (0.56%) AEs of grade 3 reported and 37 (0.25%) AEs of grade 4.

Among the 39 patients, the median response was 7 days (IQR 6.25-8.75) for patients whose target compliance was 7 days (chemotherapy or immunotherapy), and 7 days (IQR 7-16) for patients whose target compliance was 14 days (other types of treatment). Compliance was calculated for the 33 applicable patients (the other 6 patients were excluded because their participation was shorter than 30 days) and was found to be 75.8% (n=25). Of the 25 compliant patients, 92% (n=23) were still enrolled in their respective clinical trials at the end of the analysis period (March 31, 2021).

In the group with hormonotherapy, 71% (10/14) of the patients were compliant. The global tolerance of these patients was good at 92% (fraction of time where the health state was green or yellow). In the group with targeted therapy, 75% (3/4) of the patients were compliant, with a good global tolerance of 93%. In the group with combined therapies, 77% (10/13) of the patients were compliant (n=10 out of 13) with a good global tolerance of 74%.

**Figure 4:**
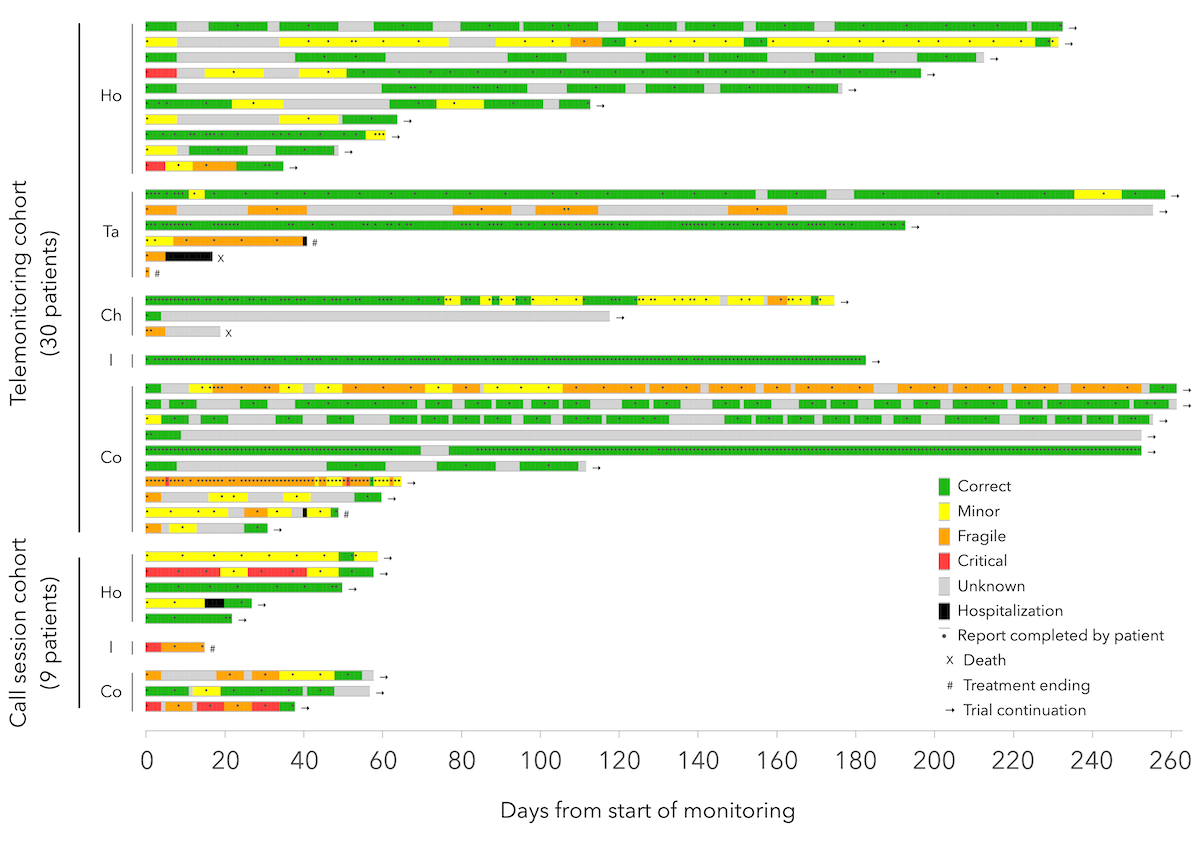
Timelines showing the health status (from the patient-reported outcomes questionnaires) and clinical events of each patient. Ch: chemotherapy; Co: combined therapy; Ho: hormonotherapy; I: immunotherapy; Ta: targeted therapy.

Six patients stopped their clinical trial because of death or disease progression. Moreover, 5 unscheduled hospitalizations were recorded during the course of this study, 2 related to AEs and 3 due to disease progression (1 is not represented in Figure 4, as it happened after the end of the analyzed timeline) (Figure 4).

### Satisfaction

**Figure 5:**
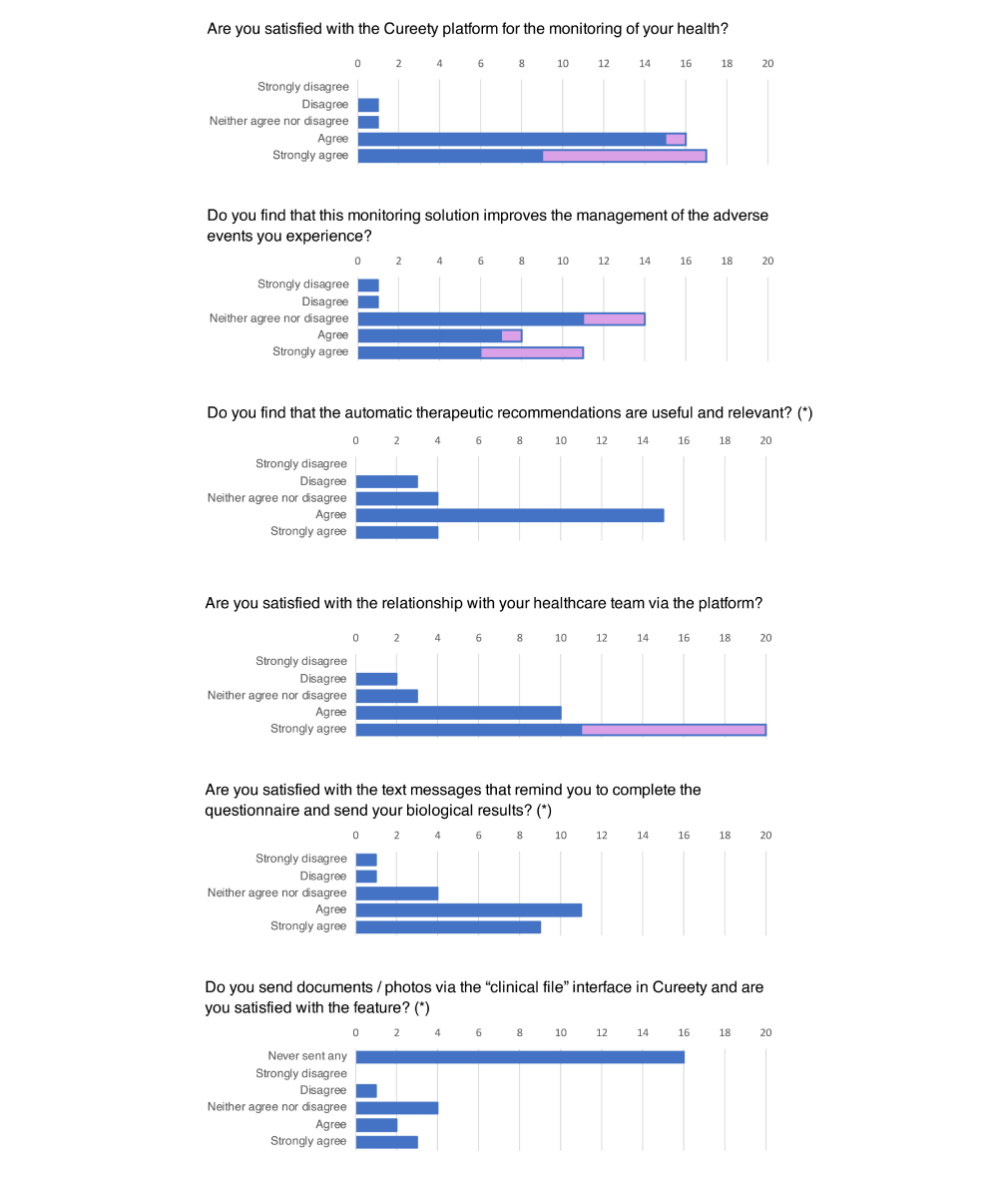
Satisfaction questionnaire results. Asterisks (*) indicate the question was only asked to patients in the telemonitoring cohort.

When prompted, 35 patients completed the satisfaction questionnaire (Figure 5), including all the patients in the “call session” cohort (9/35, 26%). The answers show that 94% (n=33) were satisfied with the monitoring platform, including 51% (n=17) who were very satisfied, and 54% (n=19) estimated that this tool improved the management of their AEs. Additionally, 85% (n=30) of the patients were satisfied with their relationship with their health care team, particularly via the platform, including 66% (n=20) who were very satisfied.

## Discussion

### Principal Findings

This connect-patient-to-doctor study is to our knowledge the first study evaluating, via a connected platform, the remote monitoring of cancer patients who are included in clinical trials.

Remote monitoring has already been shown to benefit the management of patients with chronic pathologies such as diabetes, psychiatric and cardiovascular diseases, as well as cancer [8-11]. The benefit is not just at the clinical level but is also medico-economic [11,12]. Kim et al [11] evaluated the impact of remote monitoring versus standard care in the management of patients with type 2 diabetes. In this meta-analysis, 6855 patients were included. Telemonitoring was associated with a significant decrease in glycated hemoglobin levels compared with usual care (weighted mean difference -0.42%, 95% CI -0.56 to -0.27).

In addition, telemedicine reduces geographic inequalities in access to care. Russo et al [12] reported a benefit of telemedicine on travel time savings as well as travel costs. They noted a gain of 145 miles and 142 minutes per trip with an average savings of US $18,555 per year.

The implementation of telemedicine in our current practice is favored by the increase in the use of connected objects, with more than 90% of patients having a cell phone and 87% using the internet [13].

Telemedicine, especially telemonitoring, provides direct information on the patient’s tolerance of the treatment. This practice addresses the discrepancy in AE grading when comparing perception by the patient and interpretation by the care team [14]. It also has therapeutic and psychological benefits for the patient as well as on treatment adherence [15].

Bash et al [16] evaluated the impact of symptom monitoring in the management of 766 cancer patients and found a significant improvement in the patients’ quality of life from the monitoring (34% versus 18%, *P*<.001). Remote monitoring was also found to improve the overall survival of patients. Denis et al [17] evaluated the impact of a remote monitoring platform on the overall survival of patients with bronchial cancer, compared with the standard practice. The study showed a 68% reduction in mortality risk in patients benefiting from the remote monitoring platform (hazard ratio 0.32, 95% CI 0.15-0.67, one-sided *P*=.002).

A closer monitoring of the tolerance to treatments thus allows a better management of the patients and has a proven impact on their quality of life. Here, we demonstrated the feasibility of remote monitoring for patients included in clinical trials, with a 76% compliance rate and a high satisfaction rate (94%), all without interfering with the ongoing clinical trials. The patients continued to perform the actions required by their respective trials, in addition to the reporting of AEs via the digital platform.

Our telemonitoring platform allowed us to determine the treatment tolerance profile for each patient during the entire study. It provided therapeutic advice adapted to the grade of the reported AEs. The number of unscheduled hospitalizations observed during the study period (n=5) appeared lower than what the medical team had observed in prior years (22 during the same period of time in 2020), suggesting that telemonitoring may have a positive impact on patient management. We will need a larger dedicated study to properly determine the impact of telemonitoring on unscheduled hospitalizations.

In fact, such an impact was shown by the CAPRI (Cancérologie parcours région Ile de France [Oncology Pathway in the Ile de France Region]) study [18]. This randomized study included 609 cancer patients receiving oral therapy and compared the use of a mobile telemedicine application, combined with follow-up by nurses, with standard care. The study showed a significant decrease in unscheduled hospitalizations, at 15.1% versus 22% (*P*=.04).

Our algorithm shows the accumulated impact of each AE, weighed by their grade level, instead of just considering them independently of each other, thus better reflecting the overall state of the patients. For each clinical trial, we could then estimate the tolerance profile of the patients, as measured by the percentage of time when the health state was “green” or “yellow.” The tolerance levels were good: at 92% for patients receiving hormonotherapy, at 93% for targeted therapies, and at 74% for combined therapies.

Postel-Vinay et al [19] reported the importance of long-term monitoring of the tolerance of treatments, starting from phase I in order to better determine the recommended dose for the later trial phases. In addition, the AEs and their grades seem to be predictive factors of the treatment efficacy. Socinski et al [20] recently evaluated immune-related adverse events (irAEs) in a pooled analysis treatment in patients with metastatic non–small cell lung cancer receiving chemotherapy with or without immunotherapy. They reported that patients who experienced an irAE had a gain in overall survival compared to those who did not (hazard ratio 0.69, 95% CI 0.60-0.78). This gain was mostly for patients with grade 1-2 irAE, as compared to patients with grade 3-4, with a median overall survival of 33 months (hazard ratio 0.72, 95% CI 0.59-0.89) and 29.9 months (hazard ratio 0.87, 95% CI 0.61-1.25), respectively.

The responses to the satisfaction questionnaire demonstrated a high level of satisfaction with the platform. Of the 26 patients who used the application for remote monitoring and who responded, 58% were satisfied, and 35% were very satisfied. Of the 9 patients in phone call sessions, 89% were very satisfied. The patients also had a very favorable opinion of the patient-care team relationship, with 86% of the patients being satisfied. This shows that the bond between the patients and their health care team was maintained, allowing for increased compliance and continuation of the clinical trials.

The remote monitoring approach also has an impact on the clinical trial data. The recurrent reporting by the patients provides a more accurate, more complete, and more frequent view of the treatment tolerance under investigation. This information is essential for the evaluation and approval of experimental treatment and is an important complement to the efficacy data.

Digital remote monitoring limits data loss and increases the clarity and accuracy of safety data during clinical trials. With a traditional approach, the reporting of AEs during a clinical trial is often incomplete or missing and is delayed. Allen et al [21] reported the limitations of the current AE reporting methods, with investigator-patient discrepancy and biases introduced by patient memory limitation.

Remote monitoring also allows to limit patient traveling to the care center, while ensuring the smooth running of the trial. Repeated travel is often a source of discontinuation of trial participation and an obstacle to patient enrollment. By reducing the need for in-person visits, remote monitoring helps reduce the duration of a trial, accelerate the collection of the results, and reduce costs [13].

The COVID-19 pandemic has strongly impacted our health system and has resulted in the interruption of many trials during the first wave. The use of e-technologies at each stage of clinical trials during such events is a clear way to modernize clinical trials and lift the obstacles that slow their implementation. These technologies can ensure remote trial approval and initiation, remote monitoring, remote visits, and the treatment of participants [22]. Of note, the entirety of the study was conducted during periods of high COVID-19 prevalence in France. Despite the pandemic, compliance with the use of the telemonitoring tool was high, which is very encouraging. By potentially reducing the risk of hospitalizations, such tools protect the patient from exposure to COVID-19 at the hospital.

These encouraging results should now be validated on a larger cohort with patients in clinical trials.

### Conclusion

Remote monitoring in clinical trials is feasible, with a high level of patient participation and satisfaction. It not only benefits patients, but also ensures the high quality of the trial, through the early management of adverse events, better knowledge of the tolerance profile of experimental treatments, and the removal of several biases that typically affect such trials. This e-technology should be deployed routinely as part of our daily practice and in our clinical trials.

## Abbreviations

AE: adverse event
CAPRI: Cancérologie parcours région Ile de France
CTCAE: Common Terminology Criteria for Adverse Events
ePRO: patient-reported outcomes questionnaire
irAE: immune-related adverse event
ISO: international information security standard
PARP: poly adenosine diphosphate-ribose polymerase
PRO: patient-reported outcomes
